# Angiotensin II represses *Npr1* expression and receptor function by recruitment of transcription factors CREB and HSF-4a and activation of HDACs

**DOI:** 10.1038/s41598-020-61041-y

**Published:** 2020-03-09

**Authors:** Kiran K. Arise, Prerna Kumar, Renu Garg, Ramachandran Samivel, Hanqing Zhao, Krishna Pandya, Christian Nguyen, Sarah Lindsey, Kailash N. Pandey

**Affiliations:** 1 Department of Physiology, Tulane University Health Sciences Center, School of Medicine, New Orleans, LA 70112 USA; 2 Department of Pharmacology, Tulane University Health Sciences Center, School of Medicine, New Orleans, LA 70112 USA

**Keywords:** Genetics, Molecular biology, Physiology

## Abstract

The two vasoactive hormones, angiotensin II (ANG II; vasoconstrictive) and atrial natriuretic peptide (ANP; vasodilatory) antagonize the biological actions of each other. ANP acting through natriuretic peptide receptor-A (NPRA) lowers blood pressure and blood volume. We tested hypothesis that ANG II plays critical roles in the transcriptional repression of *Npr1* (encoding NPRA) and receptor function. ANG II significantly decreased NPRA mRNA and protein levels and cGMP accumulation in cultured mesangial cells and attenuated ANP-mediated relaxation of aortic rings *ex vivo*. The transcription factors, cAMP-response element-binding protein (CREB) and heat-shock factor-4a (HSF-4a) facilitated the ANG II-mediated repressive effects on *Npr1* transcription. Tyrosine kinase (TK) inhibitor, genistein and phosphatidylinositol 3-kinase (PI-3K) inhibitor, wortmannin reversed the ANG II-dependent repression of *Npr1* transcription and receptor function. ANG II enhanced the activities of Class I histone deacetylases (HDACs 1/2), thereby decreased histone acetylation of H3K9/14ac and H4K8ac. The repressive effect of ANG II on *Npr1* transcription and receptor signaling seems to be transduced by TK and PI-3K pathways and modulated by CREB, HSF-4a, HDACs, and modified histones. The current findings suggest that ANG II-mediated repressive mechanisms of *Npr1* transcription and receptor function may provide new molecular targets for treatment and prevention of hypertension and cardiovascular diseases.

## Introduction

Atrial and brain natriuretic peptides (ANP and BNP) are endogenous cardiac hormones that regulate sodium excretion, water balance, and steroidogenesis, processes that are all largely directed toward reducing blood pressure and blood volume^1–4^. Both ANP and BNP are primarily synthesized in atrial myocytes and to a much lesser extent, they are synthesized in ventricular cells and stored in dense granules^1,5^. A third peptide, C-type natriuretic peptide (CNP), which is highly homologous to ANP and BNP, is predominantly present in the endothelial cells and the central nervous system^6^. ANP and BNP exhibit their major effects in diverse organ systems, including kidneys, adrenal glands, heart, vasculature, gonads, and adipose tissues^2,7–12^. The early discovery of three related natriuretic peptides (NPs) hormones, prompted the cloning and characterization of three distinct subtypes of natriuretic peptide receptors (NPRs). These NP receptors included: natriuretic peptide receptor-A (NPRA), receptor-B (NPRB), and receptor-C (NPRC), with binding characteristics of ANP and BNP to NPRA, CNP to NPRB, and all three NPs (ANP, BNP, and CNP) to NPRC^13–17^. NPRA and NPRB, both of which contain a guanylyl cyclase (GC) domain, are also referred to, respectively, as GC-A/NPRA and GC-B/NPRB. The NPRA has generally been considered to be the primary receptor of ANP and BNP, the reason being that most of the physiological effects of these hormones are triggered by rapidly activating this receptor and the generation of its intracellular second messenger cGMP^15,18–20^. The recent studies have indicated that the ANP/NPRA system also has a central role in insulin resistance, obesity, and metabolic syndromes^11,21,22^.

The expression and activation of NPRA is regulated by various hormonal agents, including angiotensin II (ANG II)^23–26^, endothelin^3,27^, and vasopressin^28^, as well as other stimuli such as osmoregulation^29,30^, autoregulation^19^, and cytokines and growth factors^29,31^. The pressure hormone ANG II exhibits the vascular constrictive effects and the retention of sodium and body fluid, thereby, increases the vascular tone and blood pressure^32,33^. The two vasoactive hormones ANP (vasodilatory and hypotensive) and ANG II (vasoconstrictive and hypertensive) antagonize each other at all levels, including biochemical, molecular, and physiological effects^34–37^. Previous studies have demonstrated that ANP antagonizes ANG II-induced contraction of vascular smooth muscle cells (VSMCs) and mesangial cells (MCs), agonist-induced Ca^2+^ accumulation, and activation of mitogen-activated protein kinases (MAPKs) and protein kinase C (PKC) in different tissues and cell types^35,38–44^. On the contrary, ANG II also antagonizes ANP-induced GC activity and intracellular accumulation of cGMP^23,35,45^. ANG II has been shown to hydrolyze ANP-induced cGMP levels probably by stimulating the calcium-activated cGMP-dependent phosphodiesterase, thereby antagonizing the ANP-induced inhibitory effects on MAPKs, PKC, and tyrosine kinase (TK) activity^23,24^. Interestingly, a recent study using *Npr3* (coding of NPRC) gene-knockout mice has indicated that NPRC, a non-guanylyl cyclase containing NP receptor prevents the progression of ANG II-dependent atrial fibrillation and remodeling^46^. This raises the possibility that the factors other than ANP/NPRA/cGMP, might also play a pivotal role in the cardiovascular protective effects involving NPRC, which needs to be further studied.

Promoter analysis of the *Npr1* gene has demonstrated the presence of ANG II-dependent negative regulatory *cis*-elements upstream of the transcription start site (TSS)^25^. However, the molecular mechanism of ANG II-dependent transcriptional repression and function of NPRA has not been elucidated. Also, the 5′-flanking region of *Npr1* promoter contains potential binding motifs for a variety of transcription factors, including Nkx-2.5, AP-4, GATA-1/2, cAMP-response element-binding protein (CREB), and heat-shock factor-4a (HSF-4a)^47^. However, the functional significance of these transcription factors in relation to *Npr1* gene expression has not been determined. In the present study, we examined the functional consequences of ANG II-stimulated *cis*-acting response elements, their binding sites, and mechanisms regulating the *Npr1* promoter activity. We demonstrated the role of CREB and HSF-4a in ANG II-mediated repression of *Npr1* transcription, expression, and physiological functions using cultured male mouse mesangial cells (MMCs) *in vitro* and intact male mouse aortic rings *ex viv*o.

## Results

### ANG II treatment dose-dependently attenuated *Npr1* gene transcription and expression

**Figure 1:**
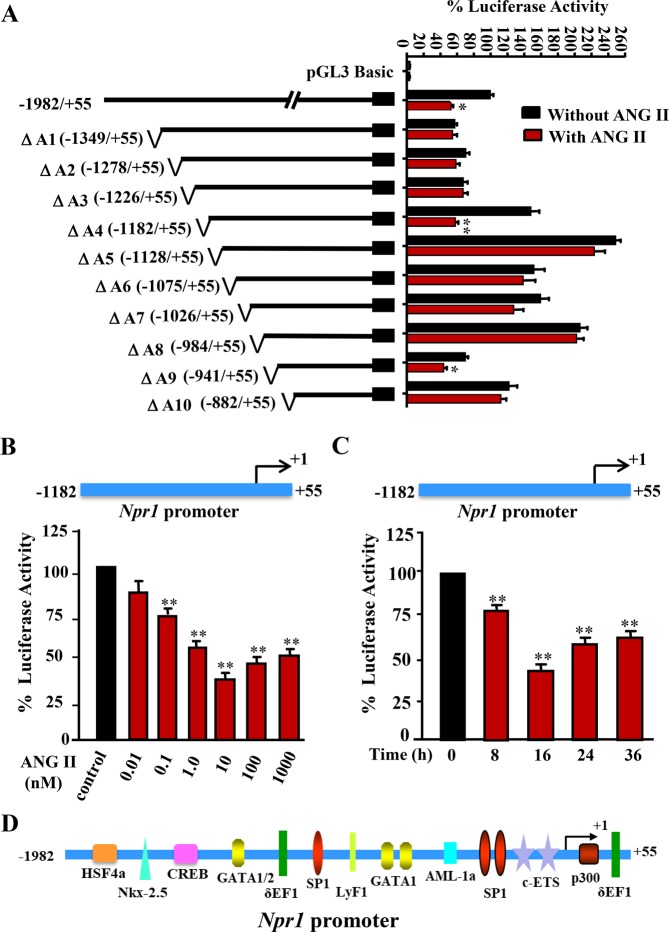
Effect of ANG II on luciferase activity of *Npr1* promoter deletion constructs in a time- and dose-dependent manner. (**A**) Different deletional constructs of the 5′-flanking region of mouse *Npr1* promoter, inserted upstream of the firefly luciferase gene. The numbers next to the schematics of *Npr1* promoter indicate the nucleotide positions for the 5′ and 3′ ends of the constructs, respectively. MMCs were transiently transfected with 1 µg of the deletion constructs and 0.3 µg of pRL-TK and after 24 h, cells were treated with ANG II (10 nM) for 16 h. (**B**) MMCs were transiently transfected with 1 µg of *Npr1* promoter construct −1182/+55 bp and 0.3 µg of pRL-TK and were treated with varying concentrations of ANG II (0.01 nM to 1000 nM) for 16 h. (**C**) Cells were transfected with *Npr1* promoter construct −1182/+55 bp and treated for varying time periods of 0, 8, 16, 24, and 36 h with ANG II (10 nM). Normalized luciferase activity is shown as a percentage of the activity of pNPRA-luc1. (**D**) Schematic representation of the putative cis-acting elements presents in the region (−1982 to +55 bp) of *Npr1* promoter. Bars represent the mean ± S.E. of 6–8 independent experiments in triplicates. *p < 0.05; **p < 0.01.

To determine the presence of ANG II responsive elements in the *Npr1* promoter, 5′ deletion constructs were sequentially analyzed for luciferase activity. The cells were transfected with deletion constructs and treated with 10 nM ANG II. As compared with untreated control cells, the constructs ΔA4 (−1182/+55 bp) and ΔA9 (−941/+55 bp) displayed significant decreases (70% and 50%, respectively) in luciferase activity (Fig. 1A). However, the constructs ΔA5 (−1128/+55 bp) and ΔA10 (−882/+55 bp) showed no corresponding reduction in *Npr1* promoter activity in response to ANG II treatment. There was a dose- and time-dependent reduction in promoter activity in ΔA4 (−1182/+55 bp) construct, with the maximum reduction occurring at a concentration of 10 nM ANG II at 16 h after ANG II treatment (Fig. 1B,C). A schematic map of *Npr1* promoter region −1982 to +55 bp containing the binding sites of various transcription factors is shown in Fig. 1D.

**Figure 2:**
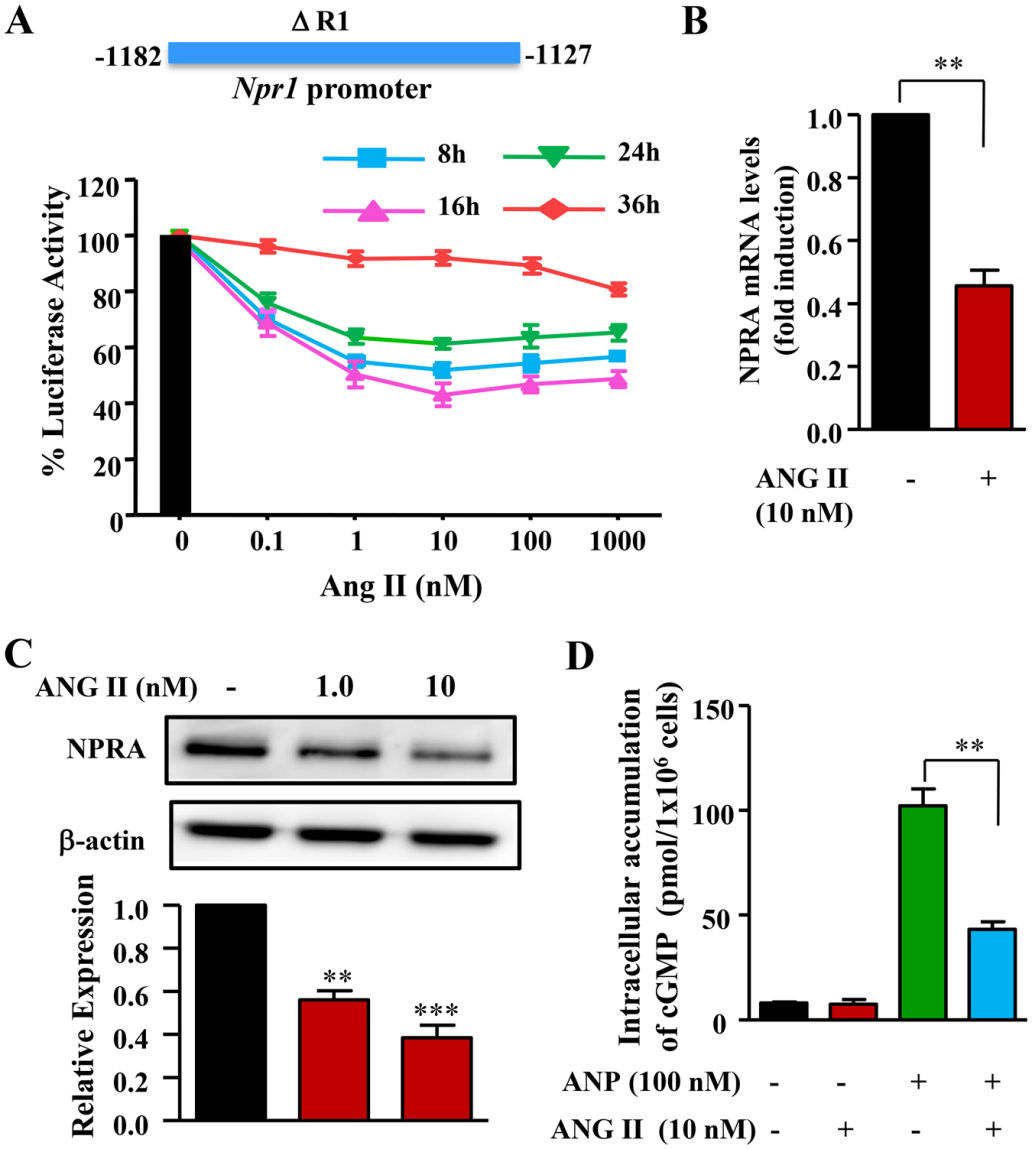
Effect of ANG II on the expression of *Npr1* mRNA and protein levels of NPRA in MMCs. (**A**) Effect of increasing concentration of ANG II with varying time periods (0, 8, 16, 24, and 36 h) of treatments on the *Npr1* promoter activity. (**B**) Effect of ANG II (10 nM) on *Npr1* mRNA expression as determined by quantitative real-time RT-PCR. (**C**) Western blot and densitometry analyses of NPRA protein (135 kDa) levels in MMCs treated with ANG II and β-actin is shown as loading control (full-length image: Supplementary Fig. 1). (**D**) Intracellular accumulation of cGMP in MMCs-treated cells  with ANG II (10 nM) and ANP (100 nM). Bar represents the mean ± SE of 6 independent experiments in triplicates. The time-course are indicated as 8 h (Blue), 16 h (Pink), 24 h (Green), and 36 h (Red) **p < 0.01; ***p < 0.001.

We next determined the activity of smaller fragments of the *Npr1* promoter exhibiting responsiveness to ANG II. As shown in Fig. 2A, the ΔR1 (−1182/−1127 bp) promoter construct exhibited dose- and time-dependent repression of luciferase activity in response to ANG II, with maximal inhibition occurring with 10 nM ANG II after 16 h of incubation. Real-time qRT-PCR assay showed 58% attenuation in *Npr1* mRNA levels in MMCs treated with ANG II as compared to untreated control cells (Fig. 2B). Similarly, there was a 60% reduction in NPRA protein levels in MMCs treated with increasing concentrations of ANG II as compared to untreated control cells (Fig. 2C). The treatment of MMCs with ANG II, showed a significant decrease in ANP-stimulated intracellular accumulation of cGMP; that decrease was almost 57% in comparison with results in unstimulated cells (Fig. 2D).

### ANG II repressed *Npr1* gene expression via AT_1_ receptor signaling

**Figure 3:**
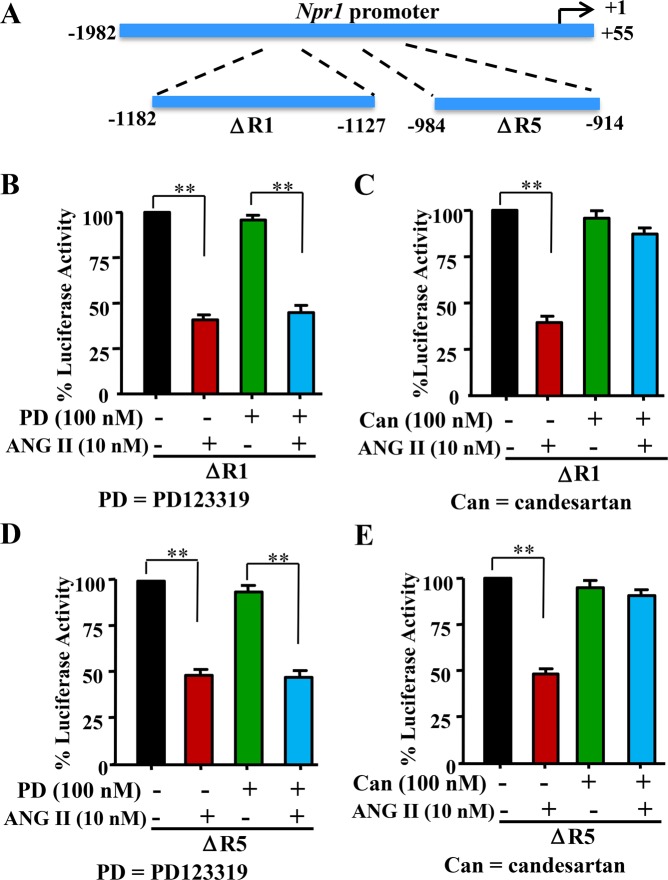
Effect of ANG II Type 1 and Type 2 receptor antagonists on ANG II-mediated transcriptional repression of *Npr1* gene promoter in the ∆R1 and ∆R5 constructs. (**A**) Schematic representation of the *Npr1* promoter constructs ∆R1 (−1182 to −1127 bp) and ∆R5 (−984 to −914 bp). MMCs were transiently transfected with 1 µg of either ∆R1 construct (**B**,**C**) or ∆R5 construct (**D**,**E**) and 0.3 µg of pRL-TK. After 24 h, the cells were treated with either 100 nM PD 123319 (**B**,**D**) or 100 nM candesartan (**C**,**E**), followed by incubation with 10 nM ANG II for 16 h. Normalized luciferase activity is shown as a percentage of the activity of an untreated group. The results are expressed as mean + SE from 7–8 independent experiments. **p < 0.01.

The results of *Npr1* promoter activity in response to candesartan, ANG II Type 1 receptor (AT_1_R) blocker, and PD 123319, an ANG II Type 2 receptor (AT_2_R) blocker, are shown in Fig. 3. A schematic map of *Npr1* promoter deletion constructs ΔR1 and ΔR5 is shown in Fig. 3A. There was a significant decrease (62%, p < 0.01) in the promoter activity of construct ΔR1 (−1182/−1127 bp) in MMCs treated with 10 nM ANG II; however, treatment with 100 nM AT_2_R blocker PD123319 had no effect on ANG II-mediated repression of *Npr1* promoter activity (Fig. 3B). Nevertheless, the repressive effect of ANG II was reversed after treatment with 100 nM of the AT_1_R blocker candesartan (Fig. 3C). Similarly, cells transfected with construct ΔR5 (−984/−914 bp) exhibited a significant reduction (50%, p < 0.01) in *Npr1* promoter activity after ANG II treatment. However, PD123319 did not reverse the ANG II effect (Fig. 3D). Also, similar to ΔR1 construct, treatment of MMCs with candesartan reversed the effect of ANG II on ΔR5 promoter activity (Fig. 3E).

### Treatment with tyrosine kinase and phosphatidylinositol-3-kinase inhibitors reversed the repressive effects of ANG II on *Npr1* promoter activity and transcription

**Figure 4:**
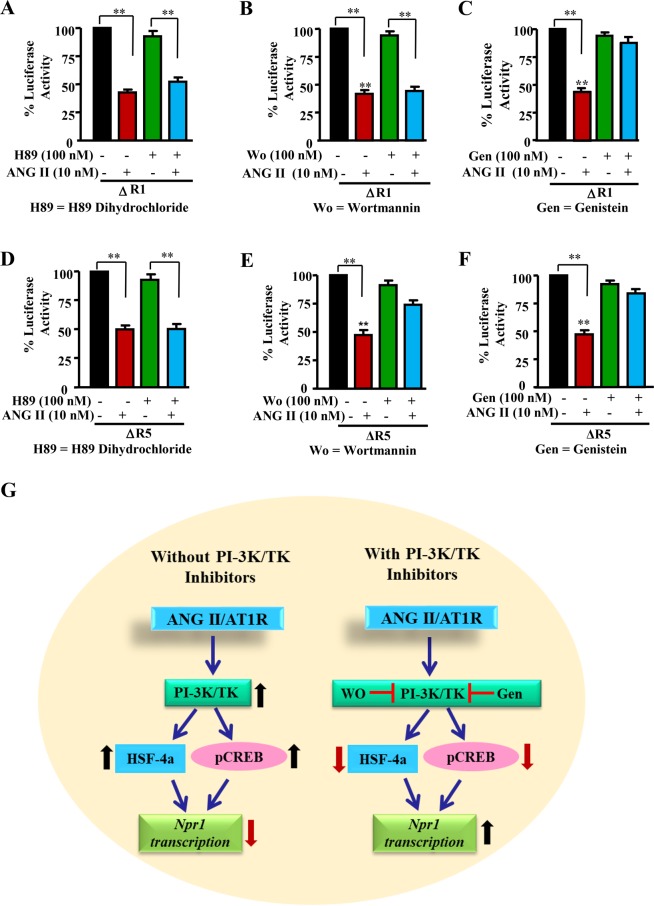
Effect of protein kinase inhibitors on ANG II-mediated transcriptional repression of *Npr1* gene promoter in ∆R1 and ∆R5 constructs. MMCs were transiently transfected with 1 µg of ∆R1 or ∆R5 construct and 0.3 µg of pRL-TK. After 24 h, cells were treated with 100 nM H-89 dihydrochloride (**A**,**D**), 100 nM wortmannin (**B**,**E**), or 100 nM genistein (**C**,**F**) and were incubated further with 10 nM ANG II for 16 h. Normalized luciferase activity is shown as a percentage of the activity of untreated groups. (**G**) Schematic representation of the effect of protein kinase inhibitors on *Npr1* gene transcription. The results are expressed as mean + SE from 7–8 independent experiments. **p < 0.01.

The promoter activity of *Npr1* in response to ANG II and protein kinase inhibitors, including inhibitors for protein kinase A (PKA), tyrosine kinase (TK), and phosphatidylinositol 3-kinase (PI-3K), are detailed in Fig. 4. There was a decrease of almost 55–60% in the promoter activity of ΔR1 construct after treatment with ANG II. However, the PKA inhibitor, H89 dihydrochloride, and the PI-3K inhibitor, wortmannin had no effect on the ANG II-mediated transcriptional activity of *Npr1* (Fig. 4A,B). Interestingly, treatment with genistein a TK inhibitor completely reversed the repressive effect of ANG II on *Npr1* ΔR1 promoter activity (Fig. 4C). Similarly, in ΔR5-transfected cells, there was 52% attenuation of *Npr1* promoter activity after treatment with ANG II alone; however, H89 dihydrochloride did not have any discernible effect (Fig. 4D). Treatment with wortmannin and genistein significantly reversed ANG II-mediated repression of *Npr1* promoter activity (Fig. 4E,F). It should be noted that wortmannin did not completely block the repressive effect of ANG II on *Npr1* promoter activity (Fig. 4E). A schematic map depicting the pathways in the presence and absence of protein kinase (PI-3K and TK) inhibitors, indicates that in the absence of inhibitors the *Npr1* transcription is enhanced; however, in the presence of inhibitors the *Npr1* transcription is repressed (Fig. 4G).

### ANG II represses *Npr1* promoter activity via recruitment of HSF-4a and CREB

**Figure 5:**
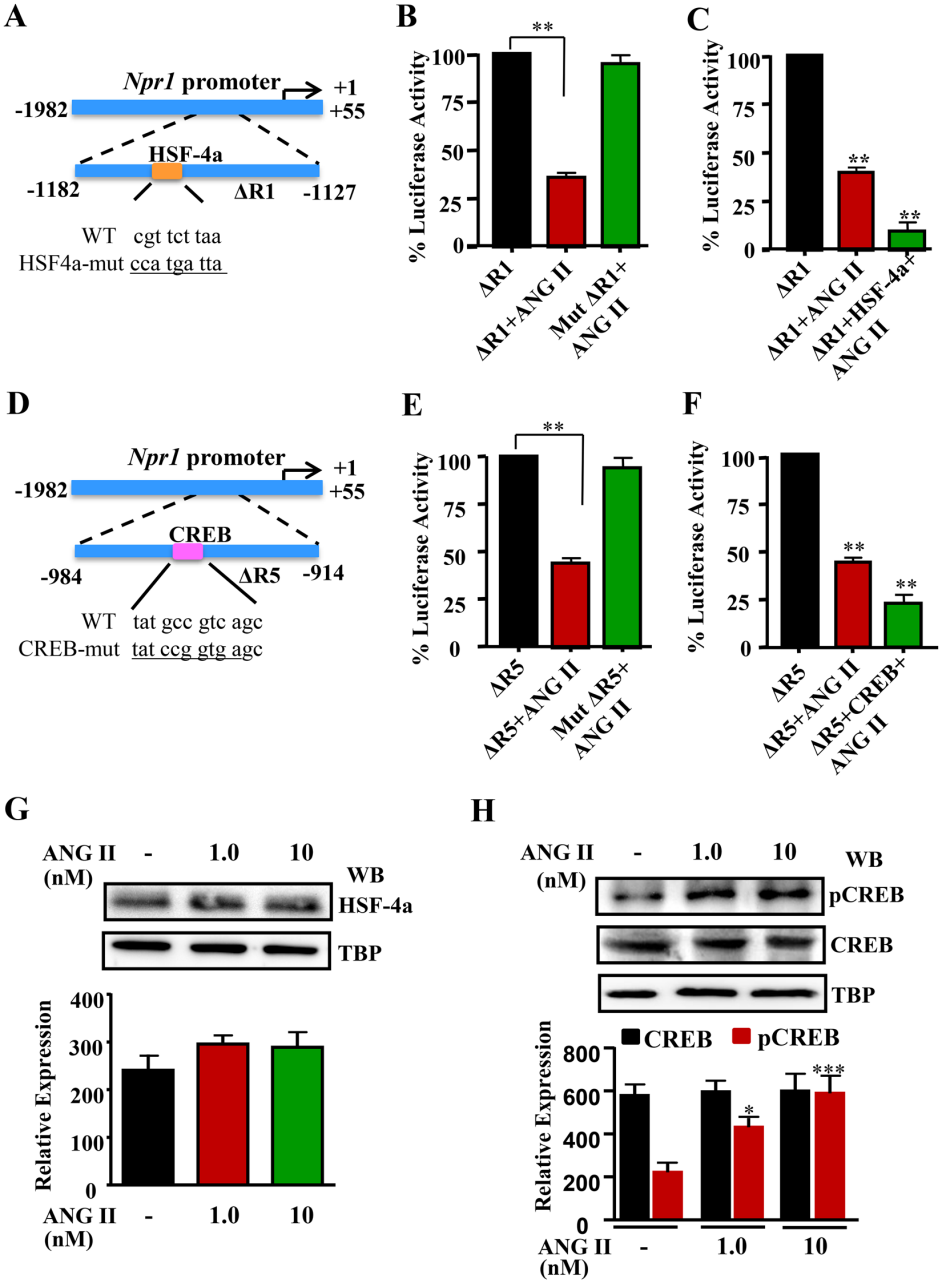
Effect of mutation in ∆R1 and ∆R5 constructs and overexpression of HSF-4a and CREB transcription factors in ANG II-mediated transcriptional repression of *Npr1* promoter. (**A**) Schematic diagram showing the sequence of the wild-type and mutated HSF-4a binding site in the *Npr1* promoter. Underlined nucleotides show the mutated sequence. (**B**) MMCs were transiently transfected with wild-type ∆R1 or mutant ∆R1 constructs, treated with ANG II for 16 h, and the promoter activity was measured. (**C**) MMCs were cotransfected with HSF-4a expression plasmid and ∆R1 promoter construct, treated with 10 nM ANG II for 16 h, and the luciferase activity was measured. Normalized luciferase activity is shown as a percentage of the activity of untreated control groups. (**D**) Schematic diagram showing the sequence of the wild-type and mutated CREB binding site in the *Npr1* promoter. (**E**) MMCs were transiently transfected with wild-type ∆R5 or mutant ∆R5 construct, treated with ANG II for 16 h, and luciferase promoter activity was measured. (**F**) MMCs were cotransfected with CREB expression plasmid and ∆R5 promoter construct treated with ANG II for 16 h, and the luciferase activity was measured. (**G**) Western blot and densitometry analysis of nuclear HSF-4a protein expression in cells treated with ANG II, for which the nuclear protein, TBP expression is shown as loading control (full-length image: Supplementary Fig. 2). (**H**) Western blot and densitometry analysis of phosphorylated nuclear CREB (pCREB) and total CREB protein expression in ANG II-treated cells for which also nuclear protein, TBP expression is shown as loading control (full-length image: Supplementary Fig. 3). Densitometry analyses of pCREB and CREB protein bands were done in the samples obtained from the same experiment and gels/blots were processed simultaneously in parallel. The results are expressed as mean + SE from 6–8 independent experiments. WB, Western blot; **p* < *0.01.

To elucidate the role of transcription factors HSF-4a and CREB, the consensus sequence cgttctt was mutated to ccatgat in ΔR1 construct (−1182/−1127 bp) and the sequence atgccgtca was mutated to atccggtga in ΔR5 construct (−984/−914 bp), as shown by schematic representation in Fig. 5A,D, respectively. Both the constructs were then transfected in MMCs to determine the *Npr1* promoter activity. As shown in Fig. 5B, after treatment with ANG II, luciferase activity was reduced by almost 65% in the wild-type ΔR1 (−1182/−1127 bp) construct. In contrast, the mutant ΔR1 (−1182/−1127 bp) construct did not show any repressive effect of Ang II on *Npr1* promoter activity. There was an almost 85% reduction in the *Npr1* promoter activity of ΔR1 construct with the overexpression of transcription factor HSF-4a (Fig. 5C). A 55% reduction of promoter activity occurred in wild-type ΔR5 (−984/−914 bp) construct in the presence of ANG II. However, the mutant construct ΔR5 (−984/−914 bp) showed no repressive effect of ANG II on *Npr1* promoter activity (Fig. 5E). On the other hand, in ΔR5-transfected cells, the overexpression of CREB reduced the ΔR5 promoter activity by almost 75% (Fig. 5F). The treatment of cells with ANG II only slightly increased HSF-4a protein expression, which was not significant as compared with untreated control cells, (Fig. 5G). However, ANG II significantly increased the phosphorylation of CREB (pCREB) in treated cells as compared with untreated control cells but did not increase the expression of total CREB protein (Fig. 5F). In the preliminary studies the transfection of only HSF-4a or CREB plasmids without ANG II did not significantly reduce the luciferase activity of *Npr1* promoter (data not shown).

**Figure 6:**
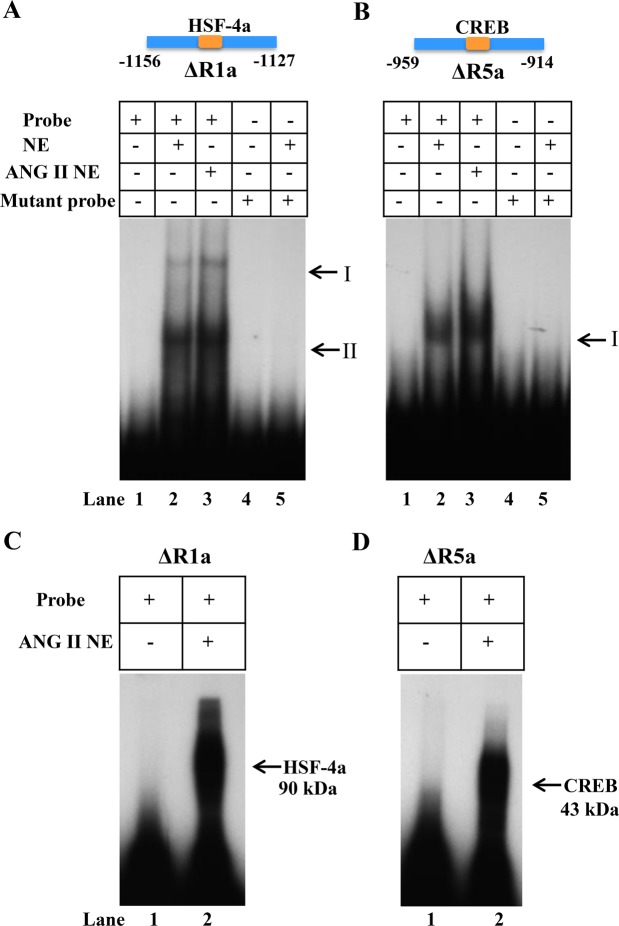
Gel electrophoretic mobility shift assay and UV crosslinking analysis of HSF-4a and CREB: Double-stranded oligonucleotides containing the consensus binding site for HSF-4a and CREB were end-labeled with [γ-p32]ATP. The DNA-protein complex was resolved from the free-labeled DNA by electrophoresis in 4% native polyacrylamide gel. (**A**,**B**) Representative autoradiographs of HSF-4a and CREB binding activity in nuclear extract from ANG II treated cells. Lane 1 shows the labeled probe without nuclear extract. Line 2 indicates the nuclear protein from untreated MMCs, lane 3 indicates nuclear protein complex binding in 10 nM ANG II-treated cells. Lane 4 shows the replacement of specific binding with 100 x excess concentrations of cold probe. Lane 5 indicates the absence of binding pattern with mutant probe, and lane 6 shows the mutant probe in the presence of nuclear extract. (**C**) UV crosslinking analysis in ANG II-treated MMCs nuclear extract. Lane 1 shows ∆R1a probe alone and lane 2 indicates binding of nuclear protein from ANG II-treated nuclear extract. (**D**) UV cross linking analysis of ANG II-treated MMCs nuclear extract. Lane 1 shows only ∆R5a probe alone and lane 2 indicates binding of nuclear protein from ANG II-treated cells. The arrows indicate the size of specific DNA-protein binding complex. EMSA and UV crosslinking images are the representative of 6–7 independent experiments.

Electrophoretic mobility shift assay (EMSA) was performed with ∆R1a (−1156 to −1127 bp) and ∆R5a (−959 to −914 bp) probes, respectively, containing HSF-4a and CREB binding sites. The ∆R1a region showed a binding pattern with band I (arrow) corresponding to HSF-4a transcription factor and an additional band II (arrow) was also observed (Fig. 6A, lanes 2 and 3). The ∆R5a region showed a binding pattern with only one band corresponding to CREB (Fig. 6B, lanes 2 and 3). In ANG II-treated nuclear extract the binding in both regions was markedly enhanced, including ∆R1a and ∆R5a (Fig. 6A,B, lane 3). Lane 4 shows the inhibition of specific binding with 100 x excess concentrations of cold probe. The specificity of the transcription factor interaction was confirmed by using the mutant ∆R1 and ∆R5 probes (lane 5), which resulted in the abrogation of a specific band in the presence of nuclear extract (lane 6). UV crosslinking analysis of the ΔR1a and ΔR5a probes confirmed the molecular weight of HSF-4a corresponding to 90 kDa (Fig. 6C, lane 2), and that of CREB corresponding to 43 kDa (Fig. 6D, lane 2).

### ANG II inhibits *Npr1* transcription by increased HDAC expression and activity, as well as decreased histone acetylation

**Figure 7:**
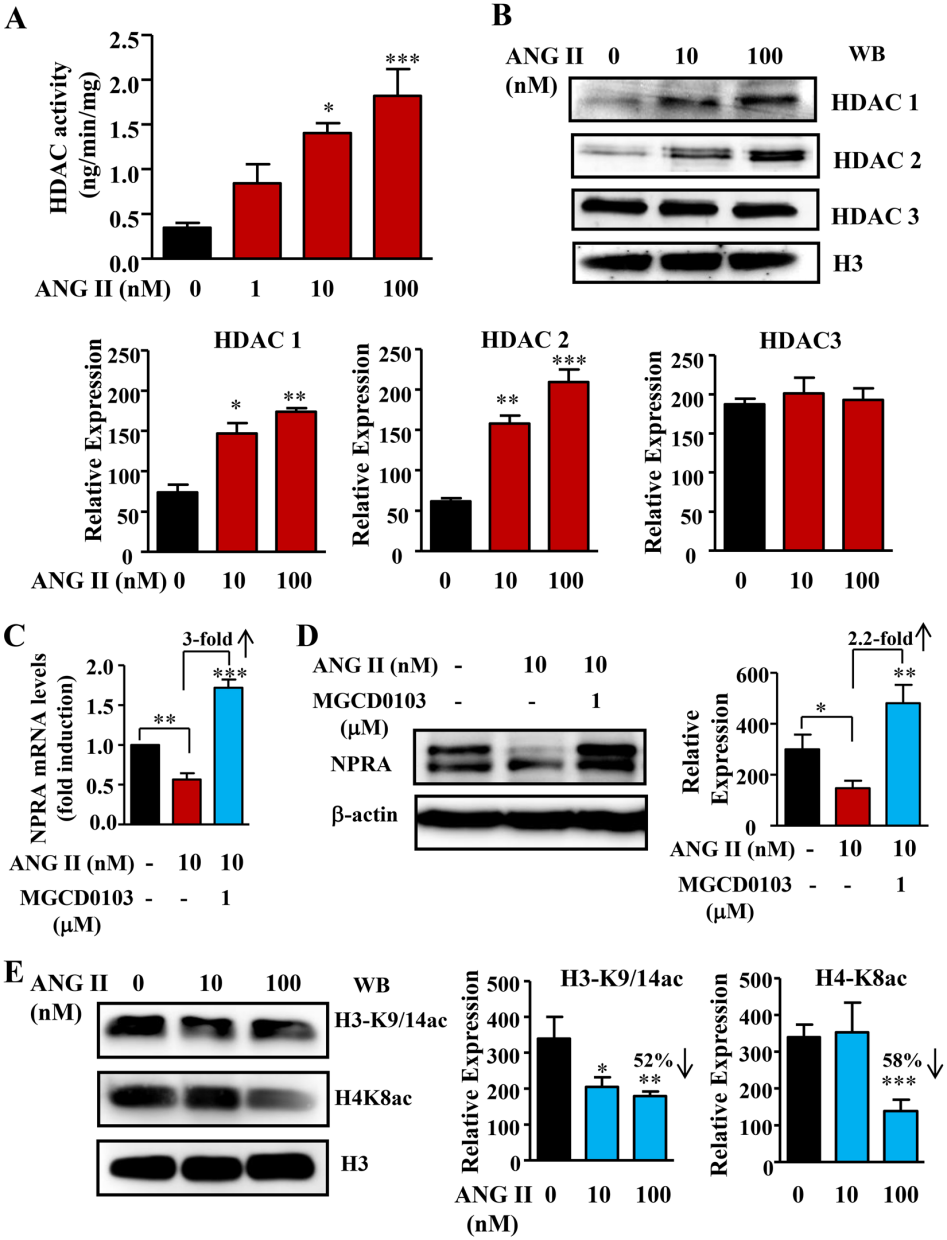
Effect of ANG II on HDAC activity and protein levels of class I HDACs in MMCs. (**A**) Effect of increasing concentrations of ANG II on total HDAC activity. (**B**) Western blot and densitometry analyses of HDAC 1, 2, and 3 protein expression in ANG II-treated cells. H3 was used as loading control (full-length image: Supplementary Fig. 4). (**C**) Effect of MGCD0103 on *Npr1* mRNA expression and (**D**) NPRA protein (135 kDa) levels in ANG II-pretreated MMCs (full-length image: Supplementary Fig. 5). (**E**) Western blot and densitometry analyses of acetylated histones H3-K9/14 and H4-K8 protein expression in ANG II-treated cells (full-length image: Supplementary Fig. 6). The results are expressed as mean ± SE from 6–8 independent experiments. WB, Western blot; *p < 0.05; **p < 0.01; **p < 0.001.

To delineate the epigenetic components involved in ANG II-mediated *Npr1* repression, we determined the effect of ANG II on histone deacetylases (HDACs) expression and activity levels of class I HDACs (HDAC1, HDAC2, and HDAC3). Treatment with increasing concentrations of ANG II augmented total HDAC activity by more than 3-fold as compared with activity in control cells (Fig. 7A). There was a significant increase in HDAC1 and 2 protein expressions in ANG II-treated cells, but no change in HDAC3 protein expression as compared with that in untreated control cells (Fig. 7B). Treatment with mocetinostat, a class I-specific HDAC inhibitor (MGCD0103) of ANG II-pretreated cells, markedly induced expression of *Npr1* mRNA and increased the protein levels of NPRA as quantified, respectively, by real time qRT-PCR and Western blot analyses (Fig. 7C,D). ANG II significantly (52%) attenuated global acetylation levels of histone H3 at lysine 9 and 14 (H3-K9/14ac) and histone H4 at lysine 8 (H4-K8ac) positions (58%) in treated as compared to untreated cells (Fig. 7E).

### Repressive effect of ANG II on *Npr1* expression and ANP-induced vasorelaxation in aortic rings

**Figure 8:**
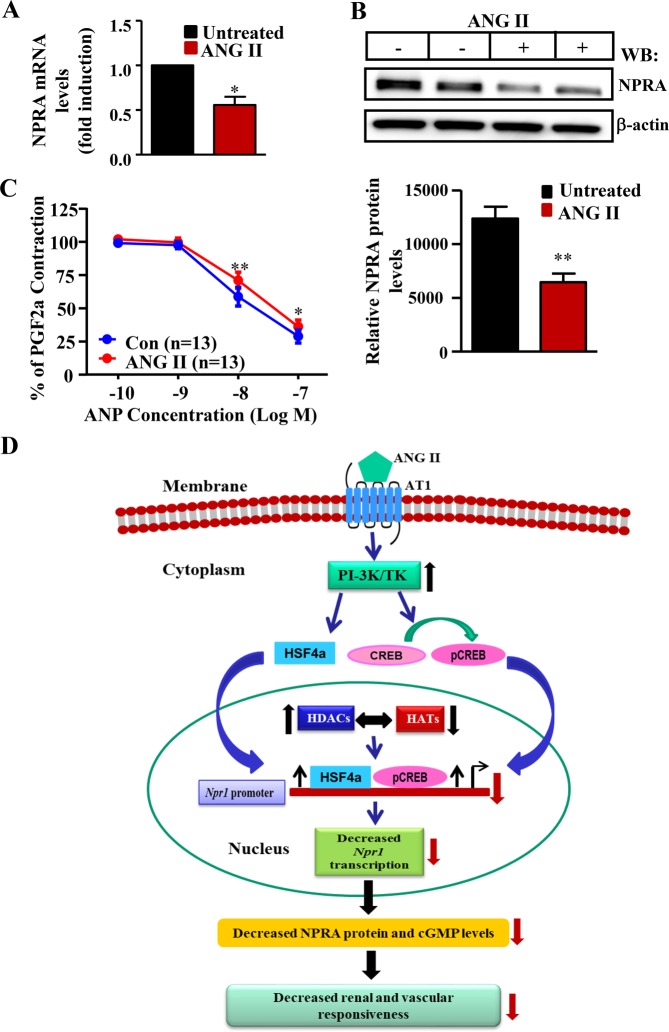
Effect of ANG II treatment on *Npr1* gene expression and ANP-induced vasorelaxation mouse of aortic rings of male mice *ex vivo*. (**A**) *Npr1* mRNA expression by real time RT-PCR and (**B**) Western blot analysis of NPRA protein (135 kDa) expression in ANG II-induced aortic rings. β-actin expression was used as loading controls (full-length image: Supplementary Fig. 7). (**C**) Vasorelaxation of aortic rings in the presence of ANP with or without ANG II treatments. (**D**) Overall, schematic model showing the antagonistic effect of ANG II on *Npr1* gene transcription and functional expression using cultured MMCs and aortic rings containing VSMCs. The model depicts that ANG II represses the *Npr1* gene transcription and expression via its AT1 receptor that enhances the PI-3K and TK signaling and recruitment of HSF-4a and CREB to the *Npr1* promoter. Furthermore, ANG II induces the HDAC activity and represses the acetylation of histones H3-K9/14 and H4-K8. The upward arrow indicates an increased HDAC activity and enhanced binding of HSF-4a and CREB to the *Npr1* promoter. The downward arrow indicates decreased *Npr1* gene transcription and reduced NPRA and cGMP levels, thereby the decreased renal and vascular responsiveness. Bars represent the mean ± SE of 8 independent experiments in triplicates. WB, Western blot; *p < 0.05; **p < 0.01.

We confirmed the effect of ANG II on *Npr1* expression by *ex-vivo* experiments using aortic rings from C57/BL6 male mice. There was a 45% reduction in *Npr1* mRNA levels in aortic rings treated with ANG II, but not in untreated control aortic rings (Fig. 8A). Incubation of denuded aortic rings with ANG II demonstrated a 50% reduction in NPRA protein levels (Fig. 8B). Treatment with increasing concentrations of ANP (IC50 = 6 × 10^−9^M) relaxed aortic rings that had been contracted with PGF2α. However, overnight treatment of aortic rings with 100 nM ANG II significantly antagonized the ANP response curve (interaction, P = 0.024). Post-hoc analysis showed significant inhibition at 10 nM (p < 0.001) and 100 nM (p < 0.05) concentrations of ANG II (Fig. 8C). In the preliminary studies for baseline control experiments, aortic rings were treated overnight in either control medium or in ANG II-containing medium and next day the rings were exposed to increasing concentrations of ANG II in the wire myograph. Rings that had been exposed to ANG II overnight did not contract in response to ANG II, indicating that a sustained tachyphylaxis occurred with down-regulation of ANG II receptors in continuous treatment protocols as shown in the Supplementary Fig. 8.

## Discussion

The results from the deletional analysis of *Npr1* promoter showed that the transcriptional activity of the core promoter in the pGL3 vector was significantly reduced in response to ANG II. Our findings provide the direct evidence in signifying the role of ANG II-response elements, CREB and HSF-4a in mediating the repressive effect of ANG II on *Npr1* transcription and functional responsiveness. The MMCs express both AT_1_ and AT_2_ receptor subtypes, which differ in their biological effects and signal transduction mechanisms^48,49^. AT_1_ receptor mediates effects such as vasoconstriction, cell proliferation, and vascular remodeling^50^, while AT_2_ receptor mediates its effect by lowering blood pressure, diuresis, natriuresis, and cell growth inhibition^51^. Both AT_1_ and AT_2_ receptors have been implicated in ANG II-dependent inhibition of ANP-stimulated GC activity of NPRA and intracellular cGMP accumulation^24,52^. However, the mechanisms involved in the mediation of the ANG II-induced effects differ for the two ANG II receptor subtypes. The stimulation of AT_1_R evokes several intracellular signals such as activation of protein kinases, including TK, PI-3K, and MAPK cascades^53,54^; on the other hand, AT_2_R activates one or several tyrosine phosphatases and MAPK phosphatase, resulting in the inhibition of specific kinases and apoptosis^55^. We investigated the inhibitory effect of ANG II on the expression of *Npr1* in the presence of receptor blockers specific to AT_1_R (candesartan) and AT_2_R (PD123319) subtypes. The AT_1_R antagonist, candesartan, but not the AT_2_R antagonist, PD123319, blocked ANG II-mediated repression of *Npr1* in both the ΔR1 and ΔR5 constructs of the *Npr1* promoter, suggesting the involvement of AT_1_ receptor subtype in this repression. Nevertheless, at higher concentrations of AT_2_R antagonist, PD123319, there was a slight repressive effect on *Npr1* promoter activity in both the ANG II responsive regions of the *Npr1* gene. Earlier, we observed that *Npr1* promoter showed repressive activity in the presence of ANG II^56^.

In the current work, we examined the involvement of protein kinases in the signal transduction pathway, mediating the repressive effect of ANG II on *Npr1* promoter. The ANG II-mediated repression of *Npr1* promoter ΔR1 construct could be blocked by genistein, a TK inhibitor, suggesting the involvement of TK in the transcriptional repression of *Npr1*. In the ΔR1 construct, there is a 100% match of the DNA sequence with a putative heat-shock element, which binds to heat-shock factors. It has been reported that HSF-4a represses basal transcription through interaction with transcription factor IIF (TFIIF), which occurs through inhibition of an early step in formation of the preinitiation complex^57^. Interestingly, ANG II causes an increase in heat-shock factors and that heat-shock protein 90 complex negatively regulates NP receptors^58,59^. Moreover, genistein has been shown to inhibit herbimycin A-induced over-expression of inducible heat-shock protein corresponding to 70 kDa^60^. Our UV cross-linking experiments and gel mobility shift assays demonstrated the formation of DNA-HSF-4a binding complex, which was enhanced in ANG II-treated cells. The novelty in the present study stems from the fact that HSF-4a is activated by ANG II, which negatively regulates *Npr1* gene transcription and receptor function. To our knowledge, this is the first report demonstrating the role of ANG II-dependent activation of TK in regulating HSF-4a in transcriptional repression of *Npr1*.

In the present study, promoter activity of the ΔR5 *Npr1* construct was repressed in the presence of ANG II; this repression could be blocked by genistein or wortmannin, suggesting the involvement of both TK and PI-3K in this pathway. The ΔR5 construct contains a putative cAMP-response element, TGCCGTCA (at −932 bp position), which is recognized by the transcription factor CREB, one of the few reported cis-elements through which ANG II has been shown to regulate gene expression. Interestingly, our results indicate that ANG II was able to activate the recruitment of CREB to *Npr1* promoter and exerted the repressive effect on *Npr1* gene transcription and function. UV cross-linking and EMSA exhibited enhanced DNA-CREB binding complex in ANG II-treated cells as compared with untreated control cells. Western blot results confirmed the enhanced phosphorylation of CREB in the presence of ANG II. It has previously been suggested that ANG II promotes the phosphorylation of CREB at Ser133 through an ERK1/2-dependent mechanism^61–63^. CREB activity has also been shown to be regulated by PI-3K/Akt signaling in Jurkat T leukemia cells treated with tumor necrosis factor-related apoptosis-inducing ligand^64^ and tyrosine kinase B/PI-3K/Akt pathway in SH-SY5Y cells treated with brain-derived neurotrophic factor^65^. Although CREB is most often described as a positive transcription factor, several reports have shown that it can also inhibit the transcriptional activity of several gene promoters such as those of c-fos and somatostatin^66,67^. It is noteworthy to mention that in our preliminary studies, the transfection of MMCs with only either HSF-4a or CREB expression plasmids alone without ANG II, did not exhibit any discernible effect on the negative repression of luciferase activity and *Npr1* transcription (data not shown). It is implicated that the hormonal signal of ANG II is required for the activation of HSF-4a and CREB to exert a repressive effect on *Npr1* promoter activity and its gene transcription.

Our present results show that ANG II treatment enhanced total HDAC activity and induced the protein expression of HDAC 1/2. Epigenetic mechanisms, including changes in histone acetylation and deacetylation have been shown to alter gene expression under various physiological and pathophysiological conditions^68,69^. Evidence suggests that ANG II treatment induces epigenetic modifications, including changes in HDACs expression and activity, which is associated with ANG II–induced tissue hypertrophy and fibrosis^70,71^. Earlier, it was shown that ANG II treatment of intact E12.5 mouse metanephroi grown *ex vivo* increased HDAC1 and decreased total acetylated histone H3 protein levels^72^. Recently, it has been shown that in ApoE gene-knockout mice with abdominal aortic aneurysms, infusion of ANG II for 4 weeks increased expression of class I HDAC1, 2, and 3, as well as expression of class II HDAC 4 and 7; it also decreased acetylation levels of H3-K18^73^. On the other hand, selective inhibition of class I HDACs led to potent suppression of ANG II-mediated cardiac fibrosis and hypertrophy by targeting cardiac fibroblasts and bone-marrow-derived fibromyocytes^71,74^. These findings are consistent with our observation that ANG II treatment repressed acetylation of H3-K9/14 and H4-K8 and enhanced HDAC activity. Our results support the notion depicting a model that ANG II facilitated the recruitment of transcription factors, CREB and HSF-4a to *Npr1* promoter via AT1R, resulting in the activation of TK and PI-3K signaling pathways, which exerted the repressive effects on *Npr1* gene transcription and function (Fig. 8D). Moreover, our model also predicts that the treatment of MMCs with HDAC inhibitor attenuated the repressive effect of ANG II on *Npr1* gene transcription, expression, and functional activity.

In conclusion, the present results demonstrate that ANG II mediates its repressive effects on *Npr1* transcription by inducing phosphorylation of CREB protein and enhancing the expression and binding of HSF-4a and CREB to the consensus sites of *Npr1* promoter. Our findings showed that the inhibitory effect of ANG II on NPRA/cGMP signaling is transduced by direct repressive effects on the *Npr1* transcription and expression via AT1 receptor, TK, and PI-3K signaling. ANG II markedly increased HDAC 1/2 protein levels and HDAC activity. The cotreatment with HDAC inhibitor reversed ANG II-mediated repression of *Npr1* transcription and function. These findings are noteworthy as they provide important insights and advance our understanding towards the action of ANG II in the repressive regulation of *Npr1* gene transcription and the ANP/NPRA/cGMP signaling pathway, which critically mediates the pathophysiology of hypertension and cardiovascular dysfunction.

## Methods and Methods

### Materials

We purchased pGL3-basic vector, pRL-TK, pGL3-control plasmids, and a dual luciferase assay system from Promega (Madison, WI). A plasmid isolation kit was bought from Qiagen (Valencia, CA). Sequence-specific oligonucleotides were purchased from Midland Certified Reagent Company (Midland, TX). Cell culture media, fetal calf serum (FCS), ITS (insulin, transferrin, and sodium selenite), and lipofectamine-2000 were purchased from Invitrogen (Carlsbad, CA). ANG II (Ang II: cat. no. H1705) was purchased from Bachem America (Prussia, PA). An RNeasy mini-kit for total RNA isolation, RT^2^ First Strand cDNA kit, and RT² SYBR Green/ROX master mix were obtained from Qiagen (Valencia, CA). An *in-vitro* Site-Directed Mutagenesis kit was purchased from Stratagene (La Jolla, CA). A direct enzyme-linked immunosorbent assay (ELISA) kit for cGMP assay was purchased from Enzo Life Sciences (Farmingdale, NY). Candesartan and PD 123319 were generously given to us by Dr. L. Gabriel Navar (Tulane University School of Medicine, New Orleans, LA). Genistein (cat. no. CAS446-72-0), wortmannin (cat. no. CAS19545-26-7), and H-89 dihydrochloride (cat. no. CAS127243-85-0) were purchased from Sigma-Aldrich Co. (St. Louis, MO). MGCD0103 (cat. no S1122) was obtained from Selleckchem (Houston, TX). HSF-4a transcription factor plasmid was given to us by Dr. Nahid Mivechi (Georgia Cancer Center, Augusta University, Augusta, GA). CREB transcription factor plasmid was a gift from Dr. Jane Reusch (University of Colorado Health Science Center and Denver VA Medical Center, Denver, CO).

### Animals

Mice used in the present studies were C57/Bl6 wild-type and produced at Tulane Vivarium. Mice were housed under 12:12 h light-dark cycle at 25 °C and fed regular chow (Purina Laboratory and tap water ad libitum as previously described^75^. Adult mice (30 g) were euthanized by deep anesthesia with isoflurane inhalation. Thoracic part of aorta was isolated and rings were prepared as earlier reported^76^. Animals were used under the protocol approved by the Institutional Animal Care and Use Committee (IACUC) at the Tulane University Health Sciences Center and were conducted in compliance with the National Institutes of Health (NIH) Guide for the Care and Use of Laboratory Animal.

### Plasmid construction in pGL3-basic

All promoter-luciferase reporter constructs were made by cloning the DNA fragments of various lengths of mouse *Npr1* gene promoter region upstream of the promoterless firefly luciferase gene in the pGL3-basic vector as previously described^25^. All the positions in the following promoter constructs are relative to TSS and were generated by polymerase chain reaction (PCR) using the pNPRA-luc1 (−1982 to +55 base pairs; bp) as a template and DNA polymerase (elongase): the ∆A1 (−1349 to +55 bp), ∆A2 (−1278 to +55 bp), ∆A3 (−1226 to +55 bp), ∆A4 (−1182 to +55 bp), ∆A5 (−1128 to +55 bp), ∆A6 (−1075 to +55 bp), ∆A7 (−1026 to +55 bp), ∆A8 (−984 to +55 bp), ∆A9 (−941 to +55 bp), and ∆A10 (−882 to +55 bp). The PCR primers used are listed in Supplementary Table S1.

### Plasmid construction in pGL3-promoter

The cloning of the smaller DNA fragments from −1182 to −914 bp of *Npr1* promoter region was done at the upstream of SV-40 promoter firefly luciferase gene in the pGL3-promoter vector as previously reported^25^. The ∆R1 (−1182 to −1127 bp), ∆R2 (−1128 to −1072 bp), ∆R3 (−1071 to −1028 bp), ∆R4 (−1026 to 986 bp), and ∆R5 (−984 to −914 bp) constructs were generated by PCR using the pNPRA-luc1 (−1982 to +55 bp) as a template and DNA polymerase (elongase). All the forward primers (F1, F2, F3, F4, and F5) contained a *Mlu*I restriction site; whereas the reverse primer contained a *Bgl*II restriction site at the 5′ ends. The PCR primers used are listed in Supplementary Table S2.

### Cell culture and hormonal treatment

MMCs were cultured in Dulbecco modified Eagle’s medium (DMEM) supplemented with 10% FCS and ITS as previously described^35^. Cultures were maintained at 37 °C in a 5% CO_2_/95% O_2_ humidified atmosphere. For all experiments, cells were used between 4 to 12 passages. To study the effect of ANG II, cells were seeded in 24-well plates at 80% to 90% confluence. The cells were washed twice with serum-free assay medium containing 0.1% bovine serum albumin (BSA) and treated with 10 nM ANG II in fresh assay medium in the absence or presence of 100 nM dihydrochloride, 100 nM wortmannin, 100 nM genistein, or 1 µM MGCD0103. The cells were harvested at the indicated time intervals and lysed essentially as described earlier^77^.

### Transient transfection and luciferase assay

MMCs were seeded in 12-well plates at a density producing ~80% confluence. After 24 h, the cells were transfected using lipofectamine-2000 reagent according to the manufacturer’s instructions, with 1 µg of test plasmid and 0.3 µg of pRL-TK carrying the renilla luciferase gene downstream of the thymidine kinase promoter, which was used as internal transfection control as earlier described^25^. The medium was changed after 24 h. The cells were harvested after 48 h by using passive lysis buffer (Promega). Luciferase activity was measured by TD 20/20 luminometer (Turner Designs, Loveland, CO) with 20 µl cell extract using a dual luciferase reporter assay system. In the transfection experiments, a pGL3-control vector containing both the SV40 promoter and enhancers was used as a positive control; the empty pGL3-basic vector was used as a negative control. The assays were performed in triplicate in 6–8 independent experiments. Results were normalized for the transfection efficiency as relative light units per renilla luciferase activity.

### Preparation of whole cell lysate and nuclear extract for Western blot analysis

Whole cell lysate and nuclear extract were prepared as described earlier^77^. The protein concentration of the lysate was measured with a Bradford protein detection kit (Bio-Rad, Hercules, CA). Western blot assay was done as previously described^35,77^. The cytoplasmic fraction (50–70 µg) or nuclear extract (40–60 µg) was mixed with sample loading buffer and electrophoresed for 2 h, then transferred to a nylon membrane. The membrane was blocked with 1x Tris-buffered saline-Tween 20 (TBST; 25 mM Tris, 500 mM NaCl, and 0.05% Tween 20, pH 7.5) containing 5% fat-free milk for 1 h, then incubated overnight in TBST containing 5% fat-free milk at 4 °C with primary antibody (1:250 dilution). The membrane was treated with corresponding secondary anti-rabbit or anti-mouse horseradish-peroxidase (HRP)-conjugated antibodies. Protein bands were developed using a SuperSignal West Femto Chemiluminescent kit and visualized using a FluorChem detection system from Proteinsimple (Santa Clara, CA). The intensity of protein bands was quantified by AlphaView software (San Jose, CA). The antibodies used in Western blot assay are listed in Supplementary Table S3.

### cGMP assay

Twenty-four hours after plating, MMCs were made serum-free for 12 h and treated with ANG II (10 nM) for another 24 h. Cells were stimulated with ANP at 37 °C for 15 min in the presence of 0.2 mM 3-isobutyl-1-methylxanthine (IBMX), washed three times with phosphate-buffered saline (PBS), and scraped into 0.5 N HCl, as previously described^19^. The cell suspension was subjected to five cycles of freeze and thaw and then centrifuged at 10,000 × g for 15 min. The supernatant thus collected was used for cGMP assays using a direct cGMP complete Elisa kit according to the manufacturer’s instructions (Enzo Life Sciences, Farmingdale, NY).

### Real-time RT-PCR analysis

Confluent MMCs were treated with or without ANG II (10 nM) in assay medium. After harvesting the cells, total RNA was extracted and 1 µg of total RNA was reverse transcribed, using a RT^2^ First Strand cDNA kit from Qiagen. Primers for amplification of *Npr1* and β-actin were from Qiagen. PCR amplification was done in triplicate in a 25-µl reaction volume using RT^2^ Real-Time SYBR Green/ROX PCR Master Mix and PCR conditions as previously described^78^. Control experiments were done with RNA samples but without reverse transcriptase. The specific primers for ß-actin gene were included in the PCR reaction mixture as an internal control.

### *In vitro* site-directed mutagenesis

The HSF-4a and CREB transcription factor mutants, ΔR1 mutant (−1182/−1127 bp) for HSF-4a and ΔR5 mutant (−984/−914 bp) for CREB, were constructed by using the *in-vitro* Site-Directed Mutagenesis kit (Stratagene). The consensus sequence of HSF-4a and CREB along with the mutant sequence is listed in Supplementary Table S4. The pGL3 plasmid with full length *Npr1* gene promoter, pNPRA-Luc1^25^, was used as a template for generating mutations. The double-stranded DNA template pNPRA-Luc1 was alkaline denatured, annealed with the mutagenic oligonucleotide and selection oligonucleotide in annealing buffer, incubated at 75 °C for 5 min, and allowed to cool slowly to 37 °C. The mutant strand was synthesized with T4 DNA polymerase and T4 DNA ligase in the presence of synthesis buffer and incubated at 37 °C for 90 min. The DNA thus synthesized was transformed in XL-10 Gold super competent cells (Stratagene) and plated in LB amp agar plates containing gene editor antibiotic mix. The probable clone was confirmed by sequencing.

### Electrophoretic mobility shift assay

The wild*-*type and mutant oligonucleotides corresponding to HSF-4a and CREB transcription factor binding sites were commercially synthesized and labeled at the 5′-end by phosphorylation of the 5′ hydroxyl ends with [γ^32^*-*P]ATP, using T4 polynucleotide kinase enzyme as previously described^79^. The radiolabeled sense oligonucleotide was annealed with the antisense unlabeled oligonucleotide in a 1:1 molar concentration by adding an equal volume of 2 x annealing buffer and incubating for 5 min in boiling water, then slowly cooling. The annealed oligonucleotides were purified on a Sephadex G-50 column. EMSA was done using ATP-labeled and annealed oligonucleotides as previously described^80^. For gel retardation, reaction mixture was prepared by adding 5*–*10 μg of nuclear extracts to 40,000*–*50,000 cpm of probe in 1x binding buffer containing 10 mM Tris (pH 7.5), 1 mM MgCl_2_, 0.05 mM EDTA, 0.05 mM DTT, 50 mM NaCl, 4% (v/v) glycerol, and 1 µg of nonspecific DNA. The reaction mixture was incubated for 30 min on ice. The DNA-protein complex was resolved from the free-labeled DNA by electrophoresis in a 4% native polyacrylamide gel at 100 V for 2 h and after drying gel was autoradiographed. For competition assays, 100-fold excess molar concentrations of unlabeled probe were added in the reaction mixture as a competitor.

### UV cross-linking analysis

To determine the molecular mass of nuclear binding proteins to the consensus sites, we did a UV cross-linking experiment. Oligonucleotide spanning the consensus site in the *Npr1* promoter was ^32^P-end-labeled and annealed for the EMSA reaction as described^80^. Binding reactions were set up as those for EMSA. After incubation on ice for 30 min, the reaction mixture was UV-cross-linked by pipetting onto parafilm and irradiated in a UV-cross linker. Samples were resolved by electrophoresis on 10% denaturing polyacrylamide gel and exposed to X-ray film for autoradiography.

### Histone purification

Total histone was extracted from ANG II-treated and untreated MMCs, using a total histone extraction kit (Epigentek, Farmingdale, NY) as earlier reported^78^. In brief, cells were harvested and suspended in 1x pre-lysis buffer, kept on ice for 10 min, and centrifuged at 10,000 × g for 1 min at 4 °C. The supernatant was removed; the cell pellet was resuspended in 3 volumes of lysis buffer, incubated on ice for 30 min, and centrifuged at 14,000 × g for 5 min at 4 °C. Balance-dithiothreitol (DTT) buffer (0.3 volumes) was added to the supernatant, which was then stored at −80 °C. The protein concentration of the eluted histone was estimated using a Bradford protein detection kit (Bio-Rad, Hercules, CA), using BSA as a standard.

### Total histone deacetylase activity assay

Total HDAC activity was measured in nuclear extracts prepared from ANG II-treated cells using a colorimetric ELISA assay kit from Active Motif (Carlsbad, CA) essentially as previously described^78^. HDAC enzyme activity was calculated by measuring the amount of HDAC-deacetylated product, which was directly proportional to HDAC enzyme activity. Absorbance was read at 450 nm. Results were calculated using a standard curve according to the manufacturer’s instructions and expressed as ng/min/mg protein.

### Treatment of aortic rings *ex vivo* with ANG II for Western blot and qRT-PCR analyses

The male mice (C57/Bl6) were euthanized by deep anesthesia with isoflurane inhalation. Thoracic aorta was isolated and aortic rings were prepared using the previously described protocol with a minor modification^31,76^. Immediately after thoracotomy, the thoracic aorta was removed and placed in cold Dulbecco’s PBS and cleaned by removing the surrounding fat and connective tissue. For experiments, the aorta was cut into 3 to 4 mm rings. After 2 h incubation in DMEM with 0.1% BSA, the aortic rings were treated with ANG II for 20 h. Aortic rings were then homogenized by sonication in lysis buffer, centrifuged, and supernatant was collected and stored at −80 °C for Western blot assay. For qRT-PCR, ANG II-treated and control rings were homogenized with 1.5-ml lysis buffer and RNA was extracted by an RNeasy mini-kit, following the manufacturer’s protocol (Qiagen, Valencia, CA).

### Aortic rings relaxation assay

Aortas were isolated and excised from C57/Bl6 male mice, cut into 2 mm rings, and incubated in DMEM containing 0.1% BSA. After 2 h, 100 nM ANG II was added to the aortic vessels. After 6 h, another dose of ANG II was added, and incubation was continued. Vessels were mounted on a wire myograph and experiments were completed as previously described^31,81^. Vessels were preconstricted with prostaglandin F2α (PGF2α) and treated with increasing concentrations of ANP. Data are expressed as percent relaxation from PGF2α contraction. For baseline control experiment, aortic rings were incubated overnight in either control media or in media containing ANG II. The next day, rings were exposed to increasing concentrations of ANG II in the wire myograph.

### Statistical analysis

Statistical analysis was done using GraphPad prism software (San Diego, CA). The results are expressed as mean ± SE. Statistical significance was evaluated by one-way ANOVA and Student *t* test. Data was also analyzed using Two-Way ANOVA. Repeated measures by both ANG II and ANP treatments and Sidak’s multiple comparisons test were used. The differences were considered significant with the probability value < 0.05.

## Supplementary information

Supplemental Information.
