# Supplementary material for: Angiotensin II represses Npr1 expression and receptor function by recruitment of transcription factors CREB and HSF-4a and activation of HDACs
Source: Sci Rep. 2020 Mar 9;10:4337. doi: 10.1038/s41598-020-61041-y (PMC7062852; doi:10.1038/s41598-020-61041-y)
Supplement: Supplementary file 1 — Supplemental Information. [file 41598_2020_61041_MOESM1_ESM.docx]

**Supplementary Material**

**Angiotensin II represses *Npr1* expression and receptor function by recruitment of transcription factors CREB and HSF-4a and activation of HDACs**

Kiran K. Arise^1^, Prerna Kumar^1^, Renu Garg^1^, Ramachandran Samivel ^1^, Hanqing Zhao^1^, Krishna Pandya^1^, Christian Nguyen^1^, Sarah Lindsey^2^, and Kailash N. Pandey^1*^

^1^Department of Physiology and ^2^Department of Pharmacology, Tulane University Health Sciences Center, School of Medicine, New Orleans, LA 70112

**Address for Correspondence:**

Kailash N. Pandey

Department of Physiology

Tulane University Health Sciences Center

1430 Tulane Ave, New Orleans, LA 70112

Tel: (504)988-1628

Fax: (504)988–2675

Email: - [kpandey@tulane.edu](mailto:kpandey@tulane.edu)

**Supplementary Table S1.** Forward and reverse primers used in making 5' and 3' deletion constructs of *Npr1* gene promoter in pGL3 basic vector. Primers are indicated in the 5' → 3' positions in the nucleotide sequence.

Position (bp) Primer Primer sequence (5' → 3')

-1349 F atc gga acg cgt ata cat atg tgt ttg tat gcc aga

-1278 F tac gga acg cgt gag aga gag aga tcc cca

-1226 F act gga acg cgt gtt atg agc cac ttg atg tag g

-1182 F atc gga acg cgt ctc tgg aag ggc aat aaa cgt tct

-1128 F atc gga acg cgt tat ctc cag ccc caa gta ccc ttt

-1074 F atc gga acg cgt ctc gct tcc aat cca ggt aga att aat

-1026 F atc gga acg cgt cat atc cct gtc tct ttt tcc agt

-984 F tc gga acg cgt gtt atg gtt att tgt cct caa aac

-941 F tc gga acg cgt ttc atc gtt atg ccg tca gca ttt

-882 F atc gga acg cgt ttt gct gac ctt aag atc ctg ttc

+55 R tac gga aga tct gcg ggt gcg cca gcg agg aaa gg

All the forward primers (F1, F2, F3, F4, F5, F6, F7, F8, F9, and F10) contained *Mlu*I restriction site, whereas the reverse primer contained *Bgl*II restriction site at the 5' ends. These restriction sites were used for cloning the amplified fragments into *Mlu*I-*Bgl*II–restricted pGL3-basic vector. All the plasmid constructs were sequenced across both the junctions to confirm the nucleotide sequence. bp, base pairs; F, forward primer; R, reverse primer.

**Supplementary Table S2.** Forward and reverse primers used in making 5' and 3' deletion constructs of *Npr1* gene promoter in pGL3 reporter vector. Primers are indicated in the 5' → 3' positions in the nucleotide sequence.

Position (bp) Primer Primer sequence (5' → 3')

-1182 F atc gga acg cgt ctc tgg aag ggc aat aaa cgt tct

-1127 R tac gga aga tct tac ttg ggg ctg gag ata t

-1128 F tac gga acg cgt tac cct ttt tta tcc ttt gac cct c

-1072 R tac gga aga tct cag gaa aac ttc caa aga g

-1071 F act gga acg cgt gct cca atc cag gta gaa tta ata g

-1028 R tac gga aga tct cca gag acc gag atg aga

-1026 F atc gga acg cgt cat atc cct gtc tct ttt tcc agt g

-986 R tac gga aga tct cac aat ata ctt taa gca c

-984 F atc gga acg cgt tat ggt tat ttg tcc tca aaa cat a

-914 R tac gga aga tct agt taa atg ctg acg gca t

The restriction sites were used for cloning of the amplified fragments into *Mlu*I-*Bgl*II–restricted pGL3-promoter vector. All the plasmid constructs were sequenced across both the junctions to confirm the nucleotide sequence and the predicted orientation. bp, base pairs; F, forward primer; R, reverse primer.

**Supplementary Table S3.** List of the antibodies used for the Western blot analysis in the current work.

**Protein Source/Catalog No. Assay Host species; Dilution**

**Monoclonal or used Polyclonal**

NPRA Genway Biotech Inc. WB Chicken; polyclonal 1:1000

#15-288-22960

(San Diego, CA)

HDAC1 SCBT Inc. # sc-8410 WB Mouse; monoclonal 1:250 (Santa Cruz, CA)

HDAC2 SCBT Inc. # sc-7899 WB Rabbit; polyclonal 1:250

(Santa Cruz, CA)

HDAC3 SCBT Inc. # sc-11417 WB Rabbit; polyclonal 1:250

(Santa Cruz, CA)

H4(K-8)ac SCBT Inc. # sc-8660-R WB Rabbit; polyclonal 1:500

(Santa Cruz, CA)

H3(K-9/14)ac SCBT Inc. # sc-8655 WB Goat; polyclonal 1:500

(Santa Cruz, CA)

H3 SCBT Inc. # sc-10809 WB Rabbit; polyclonal 1:500 (Santa Cruz, CA)

β-actin SCBT Inc. # 47778 HRP WB Mouse; monoclonal 1:5000

(Santa Cruz, CA)

CREB-1 SCBT Inc. # sc-186 WB Rabbit; polyclonal 1:500 (Santa Cruz, CA)

pCREB-1 SCBT Inc. # sc-7978 WB Goat; polyclonal 1:500

(Santa Cruz, CA)

HSTF4 SCBT Inc. # sc-19859 WB Goat; polyclonal 1:500 (Santa Cruz, CA)

SCBT, Santa Cruz Biotechnology; WB, Western blot

**Supplementary Table S4.** Forward and reverse primers used in site-directed mutagenesis of *Npr1* gene promoter in pGL3 vector. Primers are indicated in the 5' → 3' positions in the nucleotide sequence.

Oligonucleotides Position (bp) Primer sequence (5' → 3')

*Wild-type*

HSF4a -1182/-1127 ctc tgg aaggc aat aaa cgt tct taa cta ttg aac tat atc

tcc agc ccc aag ta

CREB -984/-914 tat ggt tat ttg tcc tca aaa cat ata taa taa tat tat ata

ttt cat cgt tat gcc gtc agc att taa

*Mutant*

HSF4a -1182/-1127 ctc tgg aaggc aat aaa c**ca tga tta** cta ttg aac tat atc

tcc agc ccc aag ta

CREB -984/-914 tat ggt tat ttg tcc tca aaa cat ata taa taa tat tat ata

ttt cat cgt t**at ccg gtg a**gc att taa

Underlined nucleotide sequence show wild-type and bold sequences show mutated binding sites for HSF4a and CREB transcription factors, respectively. All the plasmid constructs were sequenced across both the junctions to confirm the nucleotide sequence and the predicted orientation. bp, base pairs.


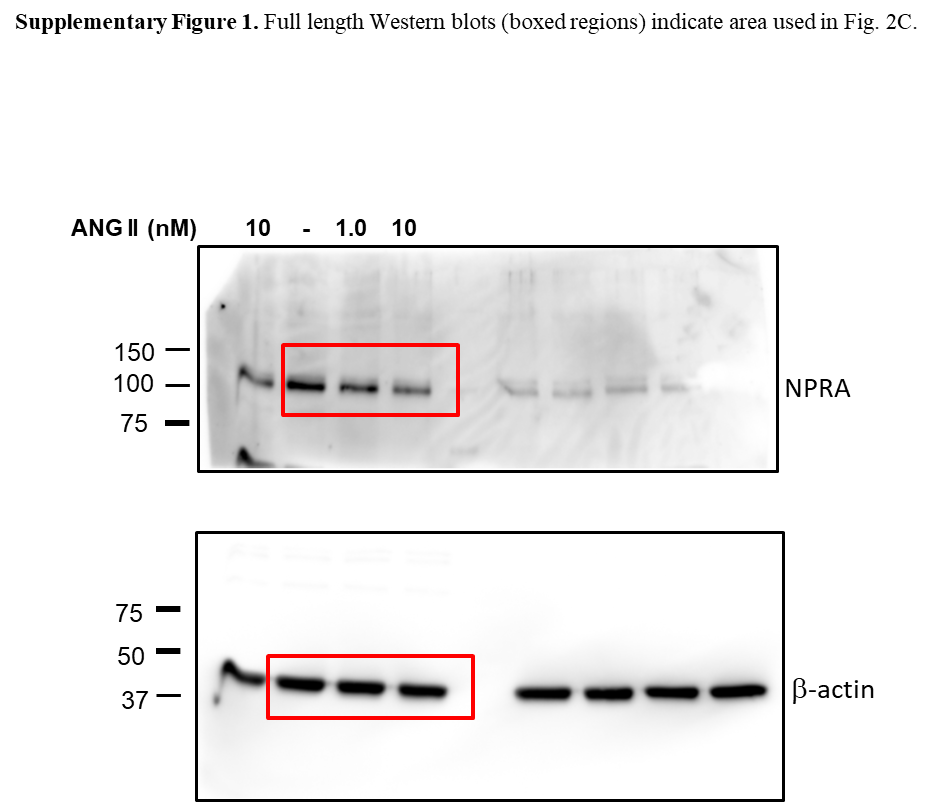


**Supplementary Figure 2.** Full length Western blots indicate the area of gel (boxed regions) used in Fig. 5G.


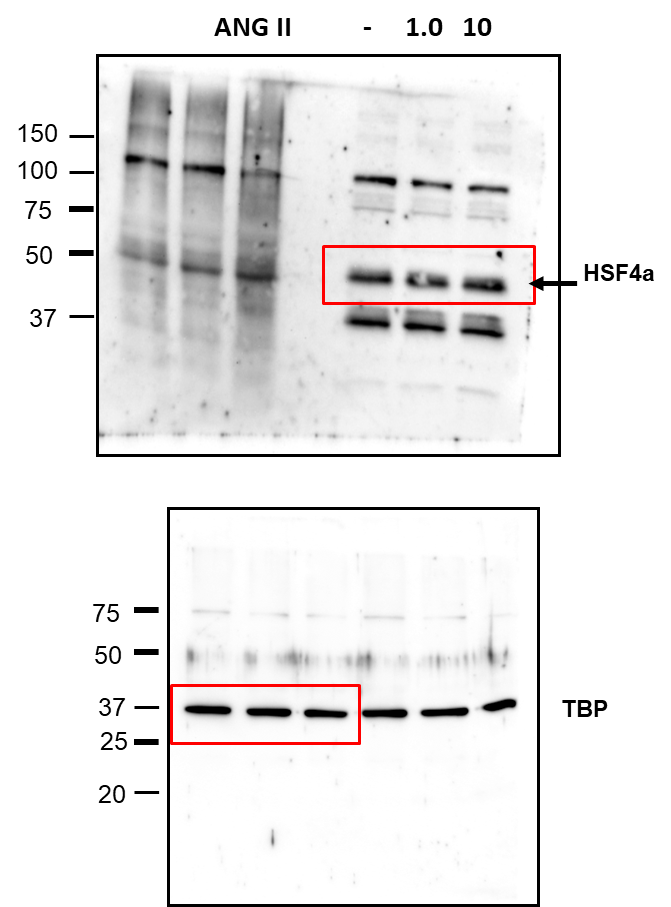


**Supplementary Figure 3.** Full length Western blots indicate the area of gel (boxed regions) used in Fig. 5H.


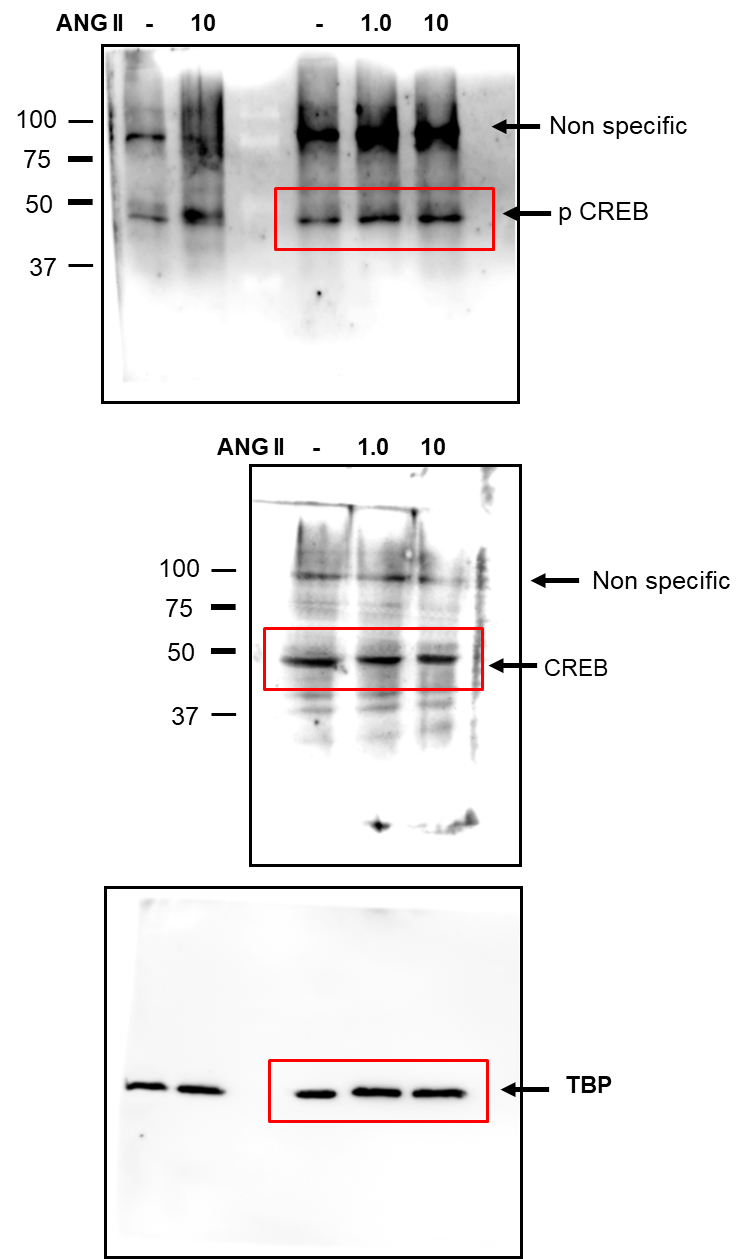


**Supplementary Figure 4.** Full length Western blots (boxed regions) indicate area used in Fig. 7B.


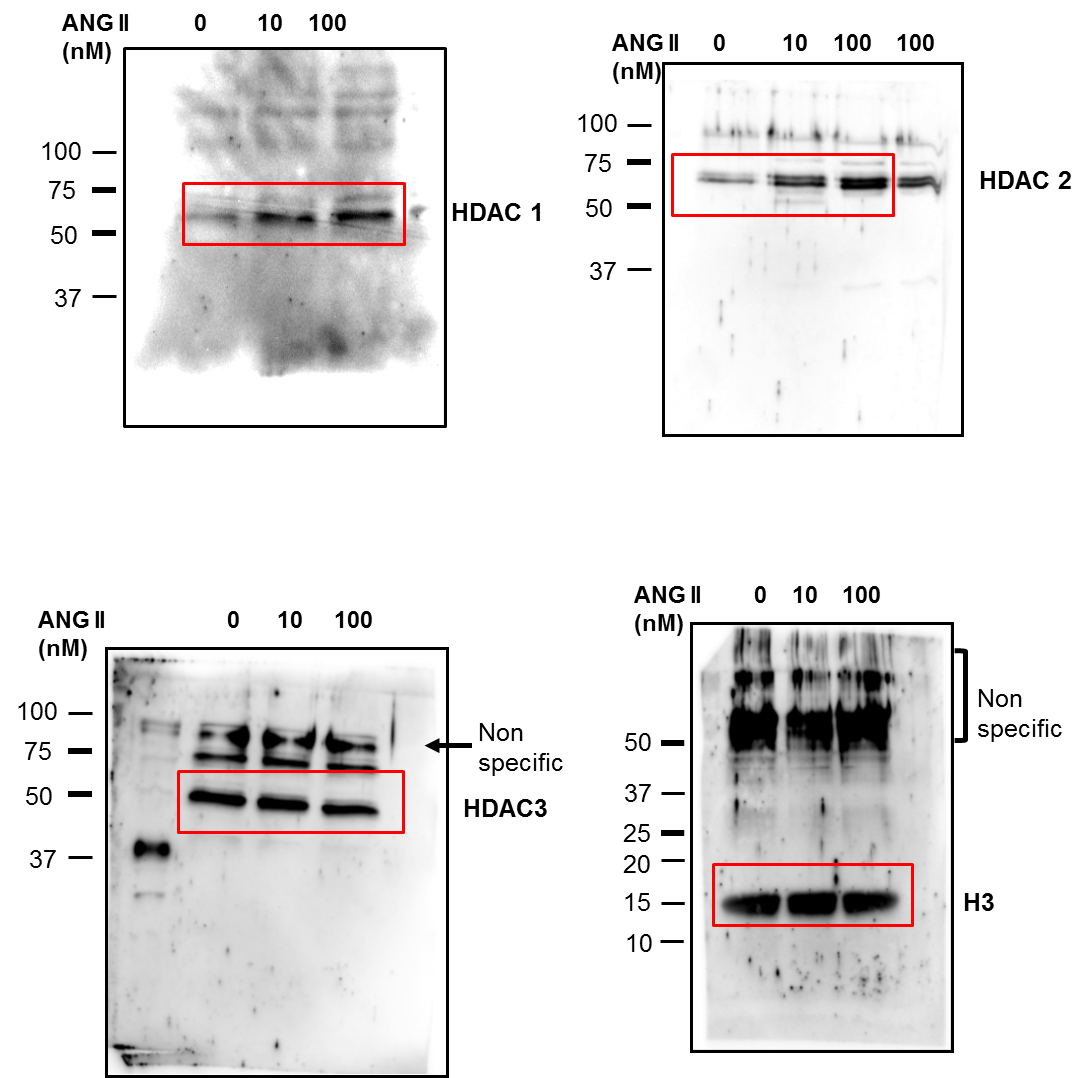


**Supplementary Figure 5.** Full length Western blots (boxed regions) indicate area used in Fig. 7D.


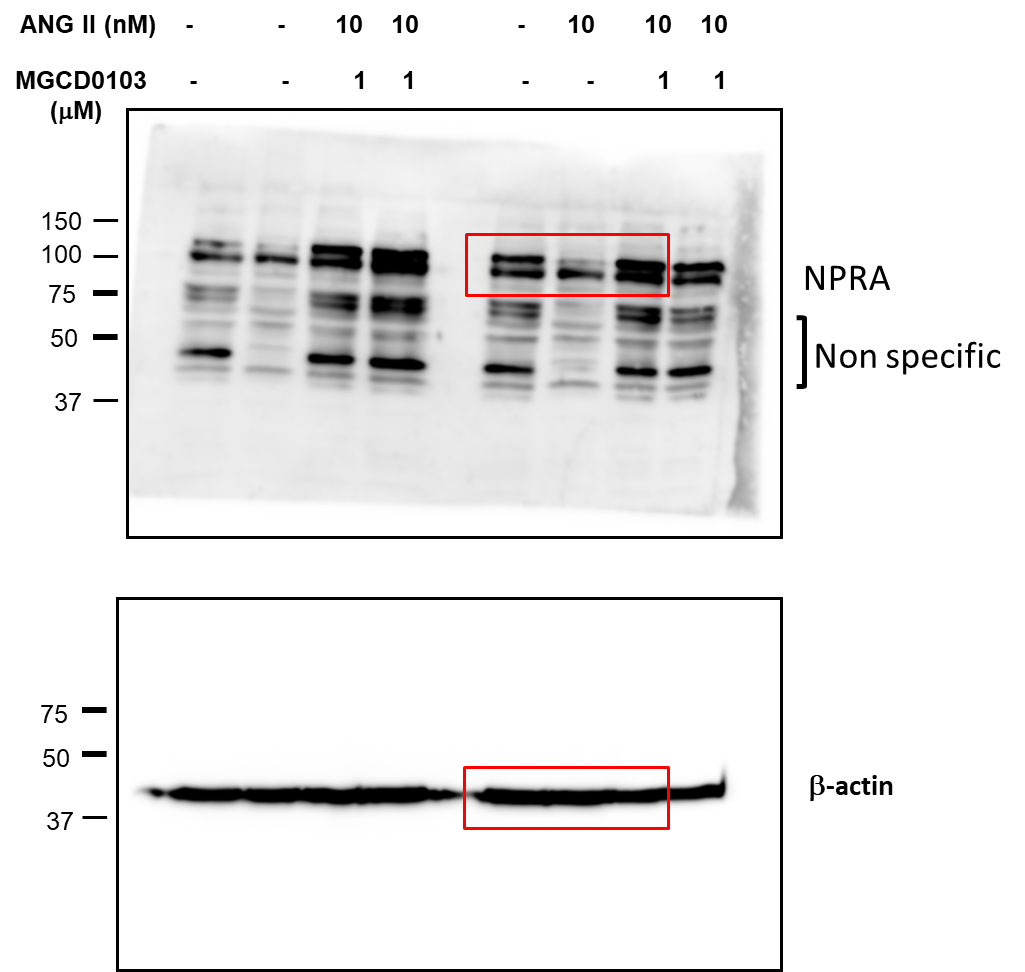


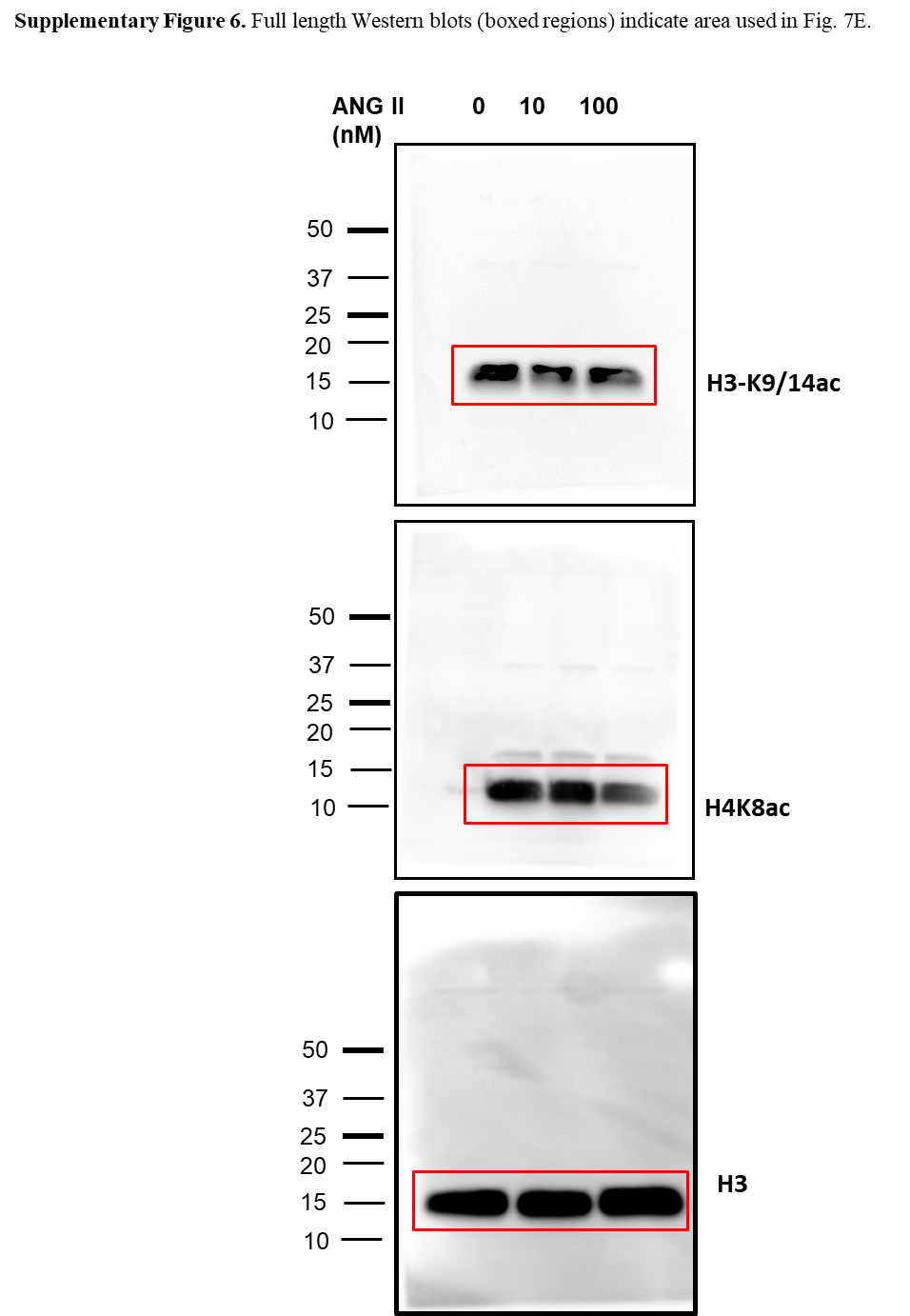


**Supplementary Figure 7.** Full length Western blots (boxed regions) indicate area used in Fig. 8B.


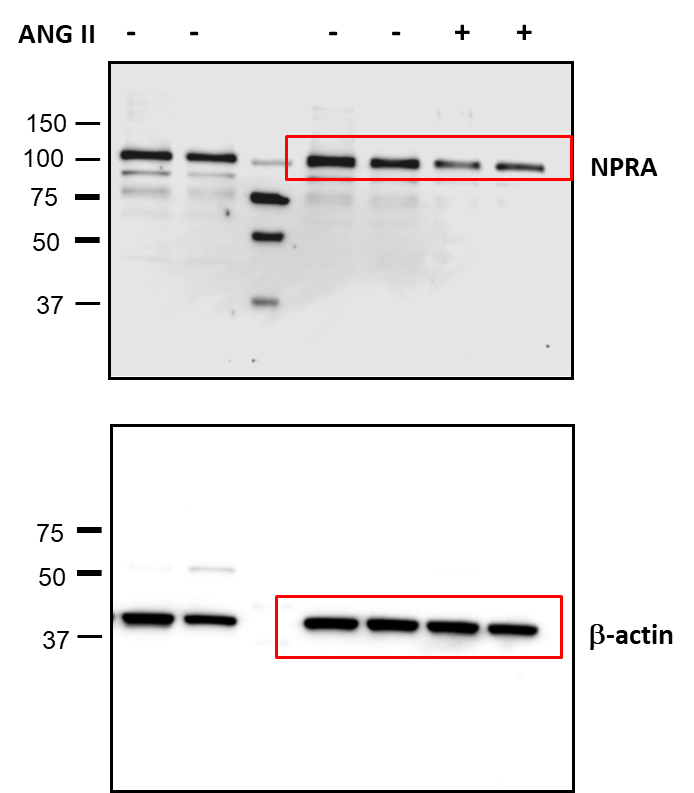


**Supplementary Figure 8:** ANG II-induced vasoconstriction in aortic rings incubated overnight.

**Result:** Aortic rings were incubated overnight in either control media or media containing 100 nM ANG II. The next day, rings were mounted on a wire myograph and exposed to increasing concentrations of ANG II. Rings that had been exposed to ANG II overnight did not contract, indicating sustained tachyphylaxis (Supplementary Fig. 8).


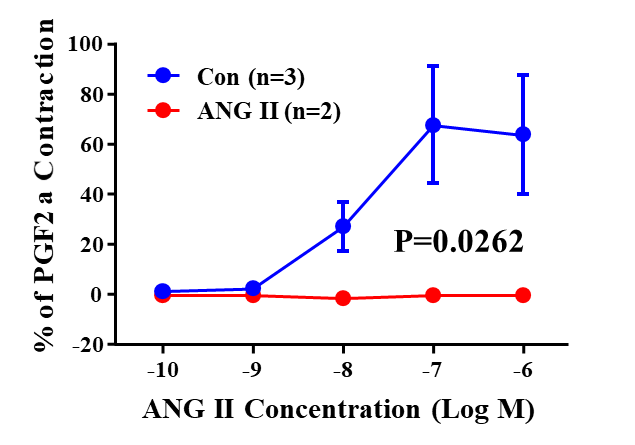


**Supplementary Figure 8:** ANG II-induced vasoconstriction normalized to PGF2α contraction. In comparison with rings incubated overnight in control media, contraction was absent in aortic rings incubated overnight in media containing ANG II, indicating sustained tachyphylaxis (2-way ANOVA, P=0.262).
